# Early initiation of guideline-directed medical therapy in patients with cardiogenic shock supported with Impella^®^: a two-center retrospective study

**DOI:** 10.1186/s12872-025-05311-5

**Published:** 2025-11-19

**Authors:** Junichiro Yokawa, Tetsuo Nishikawa, Masaya Shimojima, Miho Nakamura, Yuma Amino, Koji Sato, Yoshinao Koshida, Takumi Taniguchi

**Affiliations:** 1 Department of Intensive Care Unit, Kanazawa University Hospital, 13-1 Takaramachi, Kanazawa, Ishikawa 920-8641 Japan; 2 Department of Cardiovascular Medicine, Graduate School of Medical Sciences, Kanazawa University, Kanazawa, Japan; 3 Department of Intensive Care Medicine, Toyama Prefectural Central Hospital, Toyama, Japan; 4 Department of Anesthesiology and Intensive Care Medicine, Graduate School of Medical Sciences, Kanazawa University, Kanazawa, Japan

**Keywords:** Cardiogenic shock, Impella, Guideline-directed medical therapy, Heart failure, Intensive care unit, Cardiac intensive care

## Abstract

**Background:**

Determining the optimal timing for initiating guideline-directed medical therapy (GDMT) in survivors of cardiogenic shock remains a clinical challenge. Clinicians must balance the urgency of early intervention to prevent adverse remodeling with the risk of hemodynamic instability. This study tested the hypothesis that initiating GDMT early during the intensive care unit (ICU) stay is associated with improved 6-month clinical outcomes in this high-risk population.

**Methods:**

In a retrospective, two-center study of 42 survivors of Impella^®^-supported cardiogenic shock, patients were stratified by their simple GDMT score at ICU discharge (high-score group: score ≥ 4, *n* = 24 vs. low-score group: score < 4, *n* = 18). The primary endpoint was a 6-month composite of all-cause mortality and heart failure readmission.

**Results:**

The high-score group demonstrated significantly superior 6-month event-free survival compared to the low-score group (log-rank *p* < 0.001). Additionally, this group achieved higher GDMT scores at hospital discharge (median: 8.0 vs. 2.0, *p* < 0.001), with a greater proportion achieving a score ≥ 5 (83.3% vs. 27.8%, *p* < 0.001).

**Conclusion:**

Our findings suggest that early initiation of GDMT during ICU stabilization is associated with improved 6-month outcomes in survivors of cardiogenic shock. This hypothesis-generating study provides a rationale for future prospective trials to validate this goal-directed strategy.

**Supplementary Information:**

The online version contains supplementary material available at 10.1186/s12872-025-05311-5.

## Background

 Implementing guideline-directed medical therapy (GDMT) in the management of heart failure with reduced ejection fraction presents significant challenges within the intensive care unit (ICU), despite its central role as a therapeutic strategy. Major guidelines strongly endorse the four pillars of GDMT: renin–angiotensin system inhibitors (RASi), beta-blockers, mineralocorticoid receptor antagonists (MRAs), and sodium-glucose co-transporter 2 (SGLT2) inhibitors [1, 2]. Clinical trials have confirmed that these agents improve outcomes, particularly when administered in combination [3–12]. 

Despite these benefits, achieving comprehensive GDMT implementation is often difficult in clinical practice, especially for critically ill patients for whom concerns about hemodynamic instability and polypharmacy are considerable [13–15]. To address this gap, several scoring systems have been developed to quantify GDMT use [16–18]. Among them, the simple GDMT score proposed by Matsukawa et al.[16] is notable for its practicality. They reported that a score of ≥ 5 at hospital discharge predicted a favorable prognosis for patients with heart failure.

This evidence creates a critical dilemma for clinicians treating patients recovering from cardiogenic shock. Should GDMT be initiated cautiously during the ICU stay to suppress adverse cardiac remodelling[11], as supported by trials in stabilized acute heart failure[8], or should therapy be delayed until complete hemodynamic stability is established to avoid potential harm? The optimal timing for GDMT initiation in this high-risk population remains uncertain. Therefore, this study aimed to evaluate whether initiating GDMT during the acute phase before ICU discharge is associated with better GDMT implementation and improved 6-month outcomes in patients with cardiogenic shock supported by an Impella^®^ device. Accordingly, we investigated if early GDMT initiation, specifically before ICU discharge, was linked to greater GDMT optimization and more favorable 6-month outcomes among Impella^®^-supported patients with cardiogenic shock.

## Methods

### Study aim, design, and setting

We investigated whether GDMT initiation before ICU discharge was linked to better GDMT optimization and improved 6-month outcomes among Impella^®^-supported patients with cardiogenic shock. This retrospective, multi-center observational study was conducted in the ICUs of one university hospital and one general hospital in Japan.

### Ethics approval

The study protocol received approval from the Institutional Review Board of Kanazawa University Hospital (approval number: 2024-091, reference No. 114623). Owing to the retrospective design, the requirement for written informed consent was waived. Instead, study details were posted on the hospital website to allow patients the opportunity to opt out. As this was a retrospective study of existing clinical data, it does not meet the definition of a clinical trial, and therefore, clinical trial registration was not applicable. The study adhered to the Strengthening the Reporting of Observational Studies in Epidemiology (STROBE) statement.

### Study population

The study included all adult patients (aged ≥ 18 years) admitted to the participating ICUs between April 2019 and June 2024 who received mechanical circulatory support with an Impella^®^ device for cardiogenic shock diagnosed by the attending physicians. Patients who received Impella^®^ support solely for high-risk percutaneous coronary intervention were excluded. Patients who died during their ICU stay were also excluded because the primary exposure—GDMT score at ICU discharge—could not be assessed in these non-survivors.

### Data collection and definitions

Data were retrospectively extracted from electronic medical records. Collected variables included patient demographics, comorbidities, severity scores (Sequential Organ Failure Assessment [SOFA] and Acute Physiology and Chronic Health Evaluation [APACHE] II), Impella^®^ support details, length of ICU and hospital stay, and medications prescribed at ICU and hospital discharge.

### GDMT score calculation and patient stratification

The degree of GDMT implementation was quantified using the simple GDMT score proposed by Matsukawa et al.[16] This score ranges from 0 to 9 based on the prescription and dosing of four major drug classes (RASi, beta-blockers, MRAs, and SGLT2 inhibitors) (Supplementary Table 1).To stratify patients by early GDMT intensity, a receiver operating characteristic (ROC) curve analysis identified the optimal GDMT score at ICU discharge for predicting achievement of a prognostically relevant score ≥ 5 at hospital discharge. A score of 4 at ICU discharge was determined to be the optimal cut-off. Accordingly, patients were stratified into a high-score group (score ≥ 4) and a low-score group (score < 4).

### Outcomes

The primary endpoint was a composite of all-cause mortality and readmission for heart failure within 6 months of ICU discharge. Follow-up was conducted primarily through regional electronic medical record linkage systems. To enhance the accuracy and completeness of our follow-up data, we undertook additional efforts, including telephone interviews with patients or their families. Through this comprehensive approach, we successfully ascertained the 6-month vital status for 38 of the 42 patients (90.5%). Four patients (9.5%) were lost to follow-up, and their data were censored at the last known date of contact. Secondary endpoints included the GDMT score at hospital discharge and the proportion of patients achieving a score ≥ 5 at discharge.

### Statistical analysis

Continuous variables were assessed for normality using the Shapiro–Wilk test and visual inspection of histograms. Normally distributed data were reported as mean ± standard deviation and compared using the unpaired t-test. Non-normally distributed data were reported as median [interquatile range] and compared with the Mann–Whitney U test. Categorical variables were compared using Fisher’s exact test.

The primary outcome, 6-month all-cause mortality, was compared between the high and low GDMT score groups using the Kaplan-Meier method and the log-rank test. Data for patients lost to follow-up were censored at the last known date of contact.

As a pre-specified exploratory analysis, the association between the GDMT score at Impella^®^ removal and clinical outcomes was also assessed. Furthermore, as an exploratory subgroup analysis, we assessed whether the effect of the GDMT score was modified by the etiology of cardiogenic shock (ischemic vs. non-ischemic). A Cox proportional hazards model was used to test for a statistical interaction between the GDMT score group and the etiology. A two-sided p-value of < 0.05 was considered statistically significant. All analyses were performed using EZR (Easy R), a graphical user interface for R (The R Foundation for Statistical Computing, Vienna, Austria).

### Large language model

The authors utilized Gemini, a large language model from Google, for assistance with the text writing process, including improving the clarity and readability of the English language. The authors take full responsibility for the entire content of the manuscript.

## Results

### Patient population and stratification

**Fig. 1:**
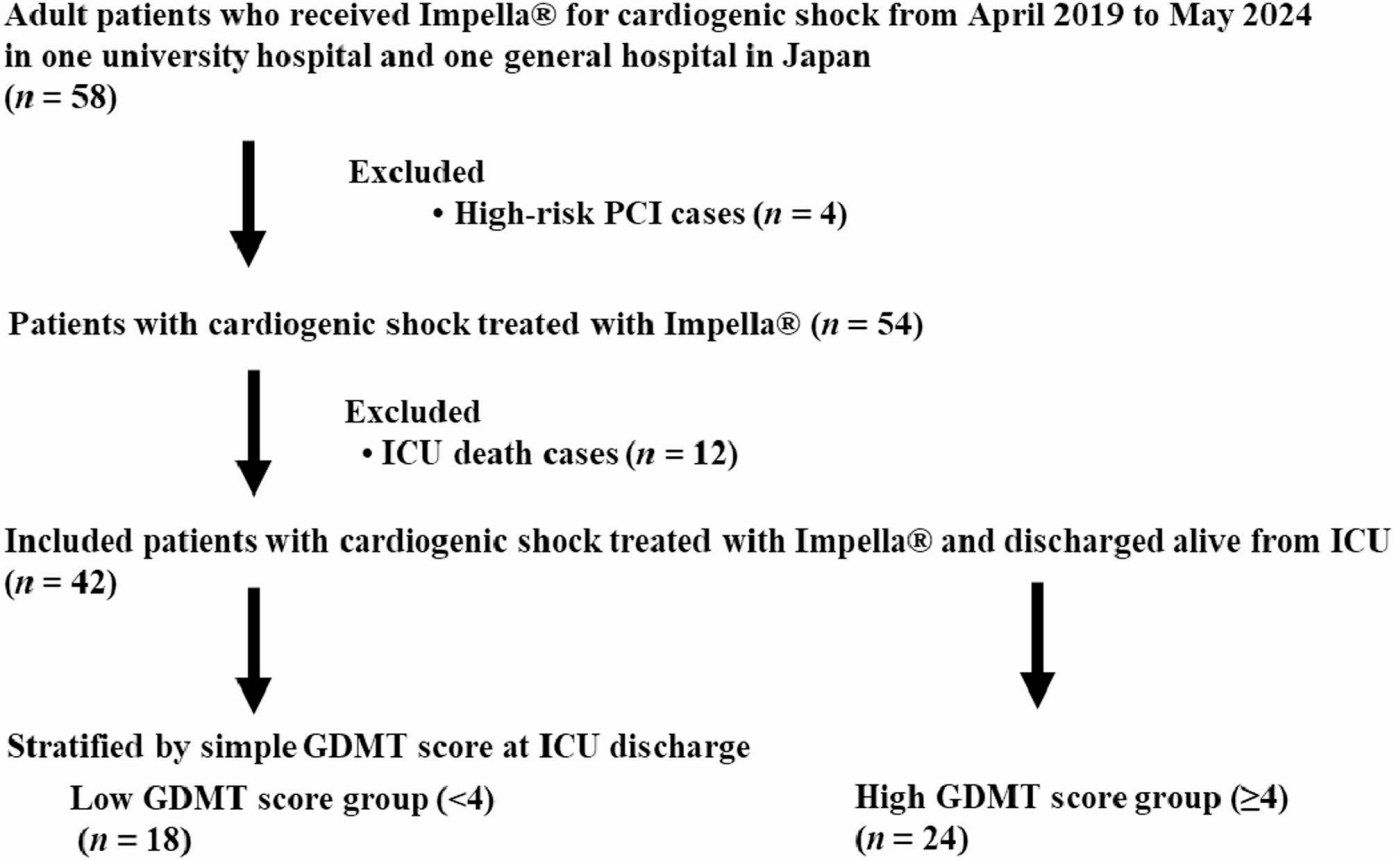
Flowchart of patient inclusion and grouping in the study

During the study period, 58 patients received Impella^®^ support for cardiogenic shock. After excluding those treated for high-risk percutaneous coronary intervention (*n* = 4) and patients who died during their ICU stay (*n* = 12), 42 patients remained for analysis (Fig. 1).

Of the 58 patients treated with Impella^®^ between April 2019 and May 2024, 4 were excluded owing to use for high-risk PCI. Among the remaining 54 patients with cardiogenic shock, 12 patients who died during ICU stay were excluded. The final analysis included 42 patients, grouped by the simple GDMT score at ICU discharge (≥ 4 vs. < 4).

**Fig. 2:**
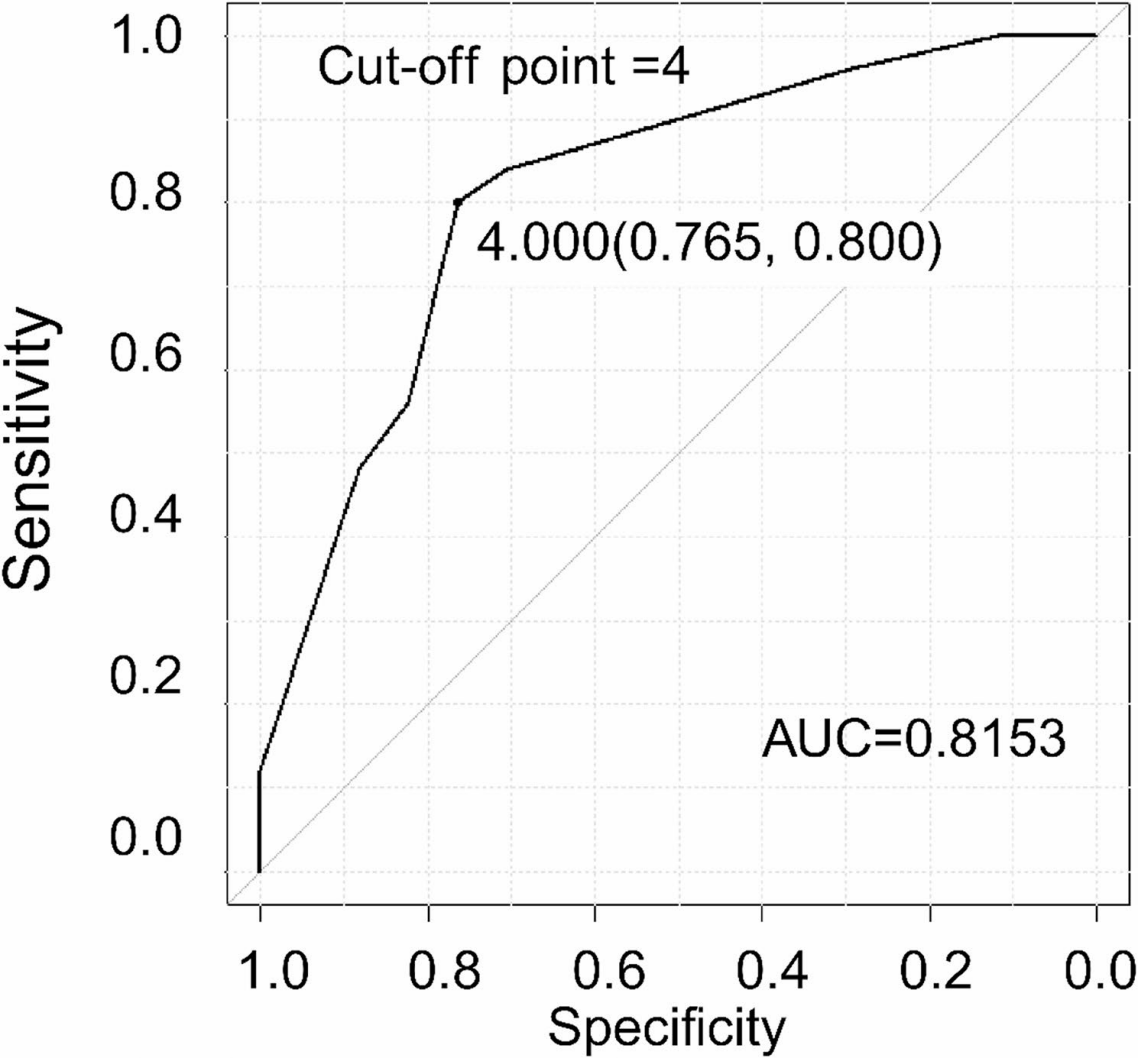
Prediction of GDMT score ≥ 5 at hospital discharge

As described in the Methods, patients were stratified by GDMT score at ICU discharge. ROC curve analysis yielded an area under the curve of 0.815 (95% CI, 0.682–0.949), identifying a score of 4 as the optimal cut-off (Fig. 2). Based on this threshold, 24 patients comprised the high-score group (GDMT ≥ 4), and 18 comprised the low-score group (< 4).

Receiver operating characteristic (ROC) curve of the GDMT score at ICU discharge for predicting the achievement of a GDMT score ≥ 5 at hospital discharge. The optimal cut-off value was 4.0, with a sensitivity of 0.800 and a specificity of 0.765. The area under the curve (AUC) was 0.815 (95% confidence interval: 0.682–0.949).

### Baseline characteristics

Baseline characteristics of both groups are shown in Table 1. Overall, the groups were similar in age, severity scores, and length of ICU and hospital stay. However, the prevalence of diabetes mellitus was significantly higher in the high-score group (58.3% vs. 16.7%; *p* < 0.05). The proportion of patients who underwent cardiac surgery was also higher in the high-score group (41.7% vs. 11.1%; *p* < 0.05). Additionally, the distribution of patients across the two participating hospitals differed significantly (*p* < 0.05).

**Table 1:** Baseline characteristics and clinical outcomes

	Low GDMT score (< 4) at ICU discharge (*n* = 18)		High GDMT score (≥ 4) at ICU discharge (*n* = 24)	*p*
Backgrounds				
Hospital A (%)		2 (11.1)	13 (54.2)	< 0.05
Hospital B (%)		16 (88.9)	11 (45.8)	
Age (years)		70.0 [64.3, 78.8]	63.5 [56.5, 73.3]	0.24
Male sex (%)		15 (83.3)	23 (95.8)	0.30
OHCA (%)		5 (27.8)	6 (25.0)	1
Surgery (%)		2 (11.1)	10 (41.7)	< 0.05
VA-ECMO (%)		8 (44.4)	8 (33.3)	0.53
PVAD				
Impella🄬 2.5/CP (%)		16 (88.9)	16 (66.7)	0.15
Impella🄬 5.0/5.5 (%)		2 (11.1)	8 (33.3)	
SOFA		9.0 [4.0, 10.0]	9.0 [6.0, 12.0]	0.26
APACHE II		21.0 [15.0, 28.0]	19.0 [12.0, 24.0]	0.39
Primary cause of cardiogenic shock				
Ischemic (%)		13 (72.2)	17 (70.8)	1
Non-ischemic (%)		5 (27.8)	7 (29.2)	
SBP (mmHg)		107.0 [92.5, 119.0]	112.0 [93.0, 138.0]	0.53
DBP (mmHg)		74.0 [55.8, 87.8]	69.50 [57.0, 97.3]	0.83
HR (bpm)		91.9 ± 43.5	102.1 ± 35.3	0.44
EF (%)		28.9 ± 12.9	29.9 ± 12.0	0.84
HFpEF (%)		1 (5.6)	3 (13.0)	0.58
HFmrEF (%)		2 (11.1)	1 (4.3)	
HFrEF (%)		15 (83.3)	19 (82.6)	
Comorbidity				
HT (%)		10 (55.6)	15 (62.5)	0.76
DM (%)		3 (16.7)	14 (58.3)	< 0.05
CKD (%)		2 (11.1)	3 (12.5)	1
Pre-hospital drug				
ACEi/ARB				0.79
< 50% of optimal dose		2 (11.1)	2 (8.3)	
≥ 50% of optimal dose		5 (27.8)	6 (25.0)	
ARNI		1 (5.6)	4 (16.7)	
β-blocker				1
< 50% of optimal dose		2 (11.1)	2 (8.0)	
≥ 50% of optimal dose		2 (11.1)	4 (16.7)	
MRA		1 (5.6)	5 (20.8)	0.21
SGLT2i		1 (5.6)	9 (37.5)	0.27
Pre-GDMT score		1.5 [0.0, 2.0]	2.0 [0.0, 4.0]	0.30
Laboratory data				
BNP (pg/mL)		187.0 [156.5, 250.0]	419.0 [189.0, 551.0]	0.21
Cr (mg/dL)		1.4 [1.0, 1.7]	1.2 [1.0, 1.5]	0.83
K (mmol/L)		4.5 [4.1, 5.0]	4.1 [3.9, 4.5]	0.14
At discharge				
Hospital days		55.0 [30.8, 79.3]	49.0 [32.8, 73.5]	0.90
ICU days		15.5 [9.8, 23.3]	13.0 [9.0, 23.5]	0.88
PVAD days		9.0 [6.3, 11.0]	7.0 [4.0, 10.5]	0.58
SBP (mmHg)		101.5 ± 20.7	109.3 ± 17.7	0.25
DBP (mmHg)		64.6 ± 14.1	63.8 ± 11.1	0.86
HR (bpm)		75.9 ± 10.1	79.4 ± 16.0	0.50
EF (%)		40.3 ± 17.1	44.6 ± 16.8	0.50
HFpEF (%)		5 (45.5)	9 (40.9)	1
HFmrEF (%)		1 (9.1)	3 (13.6)	
HFrEF (%)		5 (27.8)	10 (45.5)	
BNP (pg/mL)		384.0 [177.8, 656.0]	218.0 [151.9, 405.6]	0.27
Clinical outcomes				
Composite outcome		6 (33.3)	2 (8.3)	<0.001
HF event		1 (5.6)	2 (8.3)	1
All-cause death		5 (27.8)	0 (0.0)	<0.001
GDMT score		2.0 [0.3, 4.8]	8.0 [5.8, 9.0]	<0.001
GDMT ≥ 5		5 (27.8)	20 (83.3)	<0.001

Data are presented as mean ± standard deviation for normally distributed variables, median [interquartile range] for non-normally distributed variables, and *n* (%) for categorical variables. *p*-values represent comparisons between the low GDMT score group (< 4) and the high GDMT score group (≥ 4) at ICU discharge

*Abbreviations*: *APACHE II* Acute Physiology and Chronic Health Evaluation II, *ACEi* Angiotensin converting enzyme inhibitor, *ARB* Angiotensin II receptor blocker, *ARNI* Angiotensin receptor–neprilysin inhibitor, *BNP* Brain natriuretic peptide, *CKD* Chronic kidney disease, *Cr* Creatinine, *DBP* Diastolic blood pressure, *DM* Diabetes mellitus, *EF* Ejection fraction, *GDMT* Guideline-directed medical therapy, *HFmrEF* Heart failure with mildly reduced ejection fraction, *HFpEF* Heart failure with preserved ejection fraction, *HFrEF* Heart failure with reduced ejection fraction, *HR* Heart rate, *HT* Hypertension, *K* Potassium, *MRA* Mineralocorticoid receptor antagonist, *OHCA* Out-of-hospital cardiac arrest, *PVAD* Percutaneous ventricular assist device, *SBP* Systolic blood pressure, *SGLT2i* Sodium-glucose co-transporter 2 inhibitor, *SOFA* Sequential Organ Failure Assessment, *VA-ECMO* Veno-arterial extracorporeal membrane oxygenation

### Primary and secondary outcomes

The primary outcome was 6-month all-cause mortality. After enhancing our follow-up through supplementary telephone interviews, the vital status at 6 months was ascertained for 38 of 42 patients (90.5%).

**Fig. 3:**
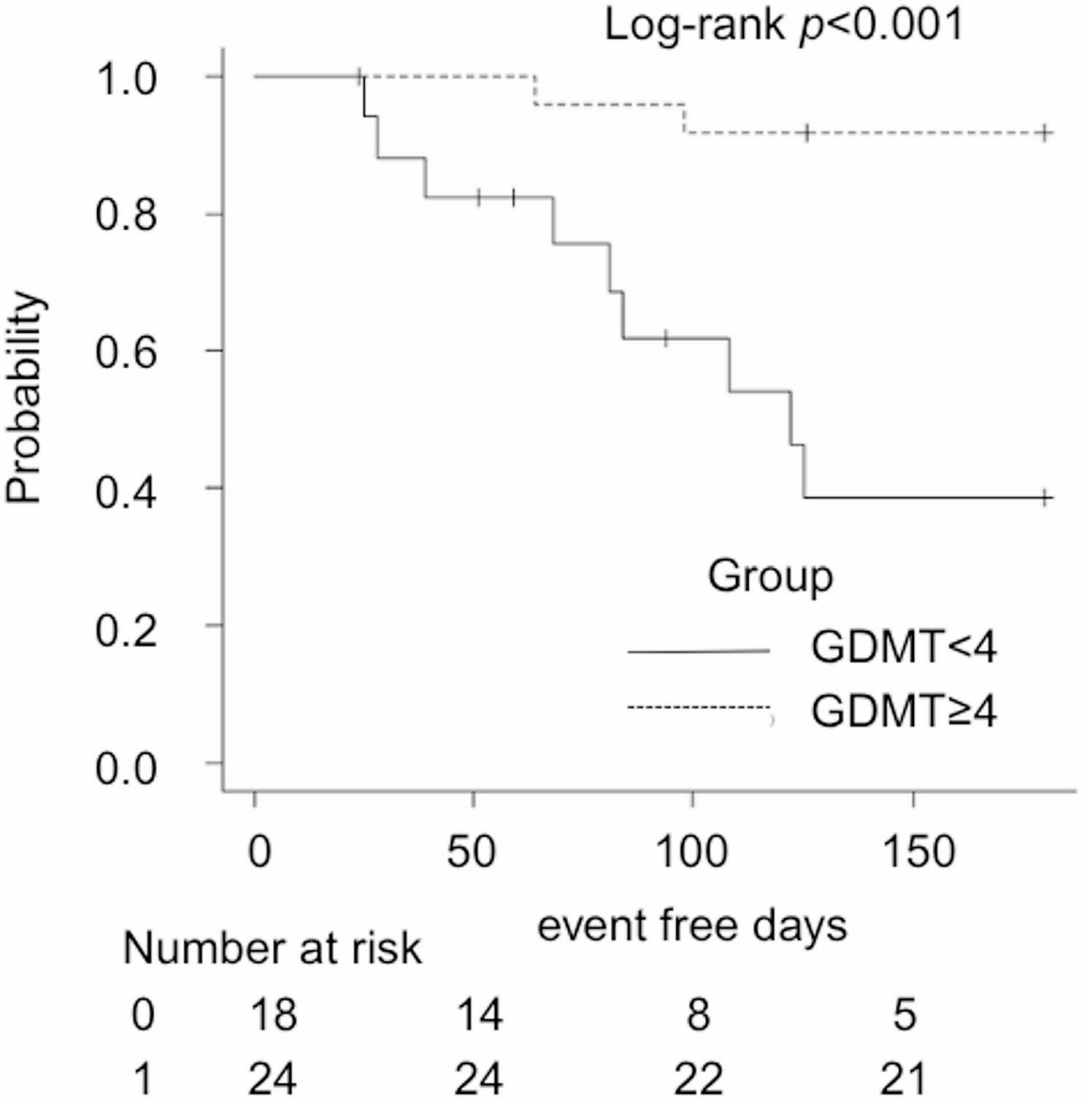
Relationship between GDMT score at ICU discharge and 6-month event-free survival

Analysis of the primary outcome demonstrated significantly better 6-month event-free survival in the high-score group. Kaplan–Meier curves showed clear separation favouring the high-score group (log-rank *p* < 0.001) (Fig. 3).

Kaplan–Meier curves for 6-month event-free survival (composite of all-cause mortality or heart failure readmission) comparing patients with a GDMT score <4 and ≥4 at ICU discharge. Patients with a higher GDMT score demonstrated significantly better outcomes (log-rank *p* <0.001).

### Secondary outcomes

**Fig. 4:**
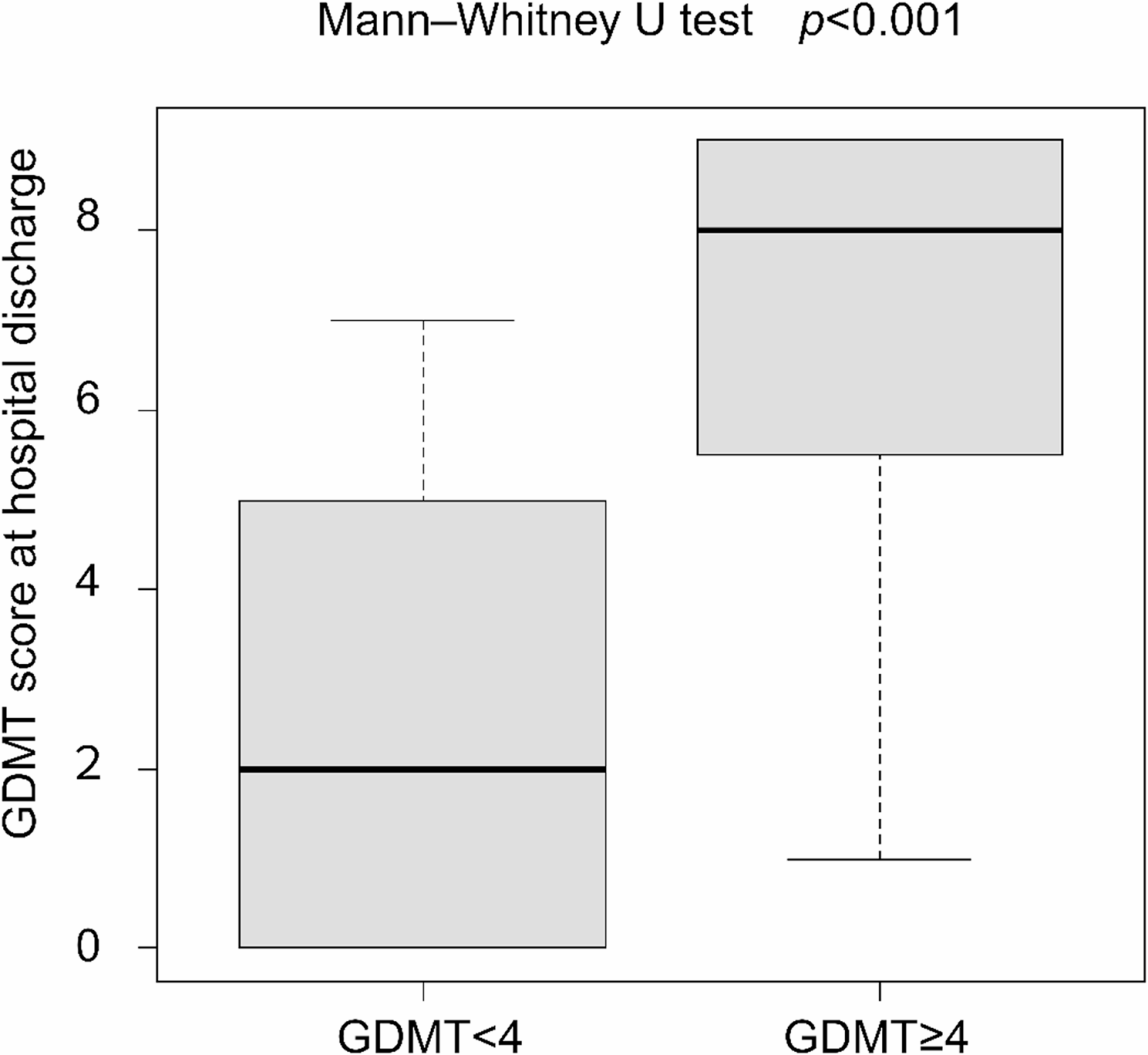
Comparison of GDMT scores at ICU discharge and hospital discharge

Secondary outcomes also favored early GDMT. Patients in the high-score group had substantially higher GDMT scores at hospital discharge (median 8.0 vs. 2.0; *p* < 0.001) (Fig. 4). Moreover, a significantly larger proportion achieved the prognostically meaningful GDMT score ≥5 at discharge (83.3% vs. 27.8%; *p* < 0.001).

Comparison of GDMT scores at hospital discharge between patients with a low GDMT score (<4) and a high GDMT score (≥4) at ICU discharge. Data are presented as boxplots showing the median, interquartile range, and minimum/maximum values. Statistical comparison was performed using the Mann–Whitney U test; *p* < 0.001.

### Exploratory analyses

In exploratory analysis, the GDMT score at Impella® removal was not associated with 6-month event-free survival (log-rank *p* = 0.67) (Supplementary Figure 1). However, a higher score at Impella® removal correlated with significantly higher GDMT scores at hospital discharge (*p* = 0.049) (Supplementary Figure 2).

 To further address the potential influence of cardiogenic shock etiology on outcomes, we performed an additional subgroup analysis stratifying patients into ischemic (*n*=30) and non-ischemic (*n*=12) groups (Supplementary Table 2). We tested for an interaction between the GDMT score group and etiology on 6-month outcomes using a Cox proportional hazards model. While this analysis was underpowered, we found no significant statistical interaction (*p *for interaction = 0.177), suggesting the beneficial association of a high GDMT score was consistent across both subgroups.

## Discussion

The principal finding of this study is that early GDMT initiation during the ICU stabilization phase is robustly associated with improved 6-month outcomes in survivors of cardiogenic shock. Our data identify a critical therapeutic window for this intervention. Specifically, this benefit was observed when GDMT was optimized to a score of ≥ 4 by the time of ICU discharge, whereas no significant association was found for GDMT initiated at the earlier timepoint of Impella^®^ removal. This dissociation suggests that while initial hemodynamic stabilization remains essential, focused initiation of GDMT during the stabilization phase within the ICU—not deferred until after ICU discharge—is a key determinant of mid-term clinical recovery. These findings challenge the notion that either urgency during peak instability or prolonged delays until full hospital stabilization are optimal strategies.

Interestingly, baseline severity scores such as the APACHE II score did not significantly differ between the groups and even showed a trend toward being lower in the high-score group. This may suggest that factors beyond initial illness severity, such as the subsequent clinical trajectory or institutional protocols, play a crucial role in determining a clinician’s ability to initiate GDMT.

Our results complement and extend findings from prior landmark studies. The STRONG-HF trial demonstrated the substantial benefit of rapid GDMT optimization in stabilized acute heart failure patients, establishing the value of early intervention [8]. Additionally, registry data from the FRENSHOCK registry and the ECLS-SHOCK trial have highlighted that GDMT implementation often remains suboptimal in shock survivors [12, 15]. Although our findings may appear to contrast with other recent trials in critically ill patients, such as the DEFENDER trial, which found no benefit of early dapagliflozin in a general population with acute organ failure [19], important differences likely explain this discrepancy. The DEFENDER trial enrolled a heterogeneous population, whereas our study focused specifically on survivors of cardiogenic shock, for whom GDMT represents the cornerstone of long-term care. Moreover, our approach evaluated a comprehensive, score-guided strategy incorporating all four GDMT pillars, rather than a single agent, initiated after initial stabilization. These observations suggest that the benefits of early GDMT may be context-specific and most relevant to post-stabilization cardiac patients managed with a goal-directed, multi-faceted approach. By demonstrating that achieving a quantifiable GDMT target (score ≥ 4) during the ICU stay is associated with significantly improved mid-term outcomes, we provide a practical, evidence-based framework for intensivists caring for this population.

The clinical implications of these findings are direct and actionable.

For the heart failure care team, this study supports transitioning from an unspecific “wait until stable” strategy to a defined approach targeting GDMT initiation during the post-weaning, pre-discharge ICU phase. Of course, this approach requires careful patient selection, as our study does not negate the clinical wisdom of avoiding GDMT in patients who remain hemodynamically fragile. A simple scoring system may offer a practical means to standardize and track this process. Critically, this framework shifts the focus from the often-debated question of which drug class to prioritize first to the more pragmatic issue of how to safely add any appropriate agent to increase the total score. This patient-tailored approach, guided by a quantitative target, may facilitate higher overall GDMT attainment than rigid, sequential protocols, as clinicians can select agents based on each patient’s hemodynamic profile and comorbidities. Nevertheless, decisions to start medications must remain individualized, balancing benefits against risks such as hypotension and bradycardia. To confirm these findings, future research should involve prospective, multi-center trials guided by standardized therapeutic algorithms targeting specific GDMT scores rather than fixed sequences.

This study has several strengths, including its novel emphasis on the timing of GDMT initiation in a well-defined, high-risk population of Impella®-supported patients with cardiogenic shock. Nonetheless, important limitations warrant acknowledgment. First, the retrospective design is a major limitation, creating an inherent risk of confounding by severity. It is highly plausible that patients with more severe illness were less able to tolerate GDMT and were therefore systematically categorized into the low-score group. Without randomization, we cannot fully account for this fundamental challenge where treatment tolerance is itself an outcome of patient severity.

Second, the modest sample size (*n* = 42) rendered the study statistically underpowered for definitive conclusions and resulted in wide confidence intervals. This limitation is particularly relevant to our exploratory subgroup analysis of ischemic versus non-ischemic etiologies, which should be interpreted with caution. Therefore, our findings should be interpreted as hypothesis-generating rather than conclusive.

Third, despite our efforts to enhance data accuracy through supplementary telephone interviews, a 9.5% loss to follow-up rate remains. This could potentially introduce selection bias if the characteristics and outcomes of the patients lost to follow-up systematically differed from those who were successfully tracked.

Finally, the composite score itself is a limitation, as it does not isolate the effects of specific drug classes. Additionally, the score reflects pharmacologic intensity but does not capture the clinical reasoning for non-prescription, such as valid contraindications or intolerance.

## Conclusions

In conclusion, despite the inherent limitations of its retrospective design and small sample size, this hypothesis-generating study offers preliminary evidence supporting a critical therapeutic window for GDMT initiation in survivors of cardiogenic shock. Our findings suggest that a flexible, score-guided strategy during ICU stabilization is associated with improved 6-month outcomes. Further prospective, adequately powered studies are warranted to confirm these observations and to establish this goal-directed approach as a new paradigm for improving prognosis in this high-risk population.

## Supplementary Information

Supplementary Material 1

## Data Availability

The data that support the findings of this study are available from the corresponding author upon reasonable request. Data sharing is subject to the approval of the Institutional Review Board of Kanazawa University Hospital and requires a signed data sharing agreement to ensure patient confidentiality.

## Abbreviations

CI: Confidence interval
GDMT: Guideline-directed medical therapy
ICU: Intensive care unit
MRA: Mineralocorticoid receptor antagonist
RASi: Renin-angiotensin system inhibitor
ROC: Receiver operating characteristic
SBP: Systolic blood pressure
SGLT2: Sodium-glucose co-transporter 2
SOFA: Sequential Organ Failure Assessment

## References

[1] Heidenreich PA, Bozkurt B, Aguilar D, Allen LA, Byun JJ, Colvin MM, et al. 2022 AHA/ACC/HFSA guideline for the management of heart failure. Circulation. 2022;145:e876–94. 10.1161/CIR.0000000000001063.35363500

[2] McDonagh TA, Metra M, Adamo M, Gardner RS, Baumbach A, Bohm M, et al. 2021 ESC guidelines for the diagnosis and treatment of acute and chronic heart failure. Eur Heart J. 2021;42(36):3599–726. 10.1093/eurheartj/ehab368. 34447992 10.1093/eurheartj/ehab368

[3] Packer M, Coats AJS, Fowler MB, Katus HA, Krum H, Mohacsi P, et al. Effect of carvedilol on survival in severe chronic heart failure. N Engl J Med. 2001;344:1651–8. 10.1056/nejm200105313442201. 11386263 10.1056/NEJM200105313442201

[4] Pitt B, Zannad F, Remme WJ, Cody R, Castaigne A, Perez A, et al. The effect of spironolactone on morbidity and mortality in patients with severe heart failure. N Engl J Med. 1999;341:709–17. 10.1056/nejm199909023411001. 10471456 10.1056/NEJM199909023411001

[5] McMurray JJV, Solomon SD, Inzucchi SE, Kober L, Kosiborod MN, Martinez FA, et al. Dapagliflozin in patients with heart failure and reduced ejection fraction. N Engl J Med. 2019;381:1995–2008. 10.1056/NEJMoa1911303. 31535829 10.1056/NEJMoa1911303

[6] Packer M, Anker SD, Butler J, Filippatos G, Pocock SJ, Carson P, et al. Effect of empagliflozin on clinical stability of patients with hfref: the EMPEROR-Reduced trial. Circulation. 2021;143:326–39. 10.1161/CIRCULATIONAHA.120.051783. 33081531 10.1161/CIRCULATIONAHA.120.051783 PMC7834905

[7] Myhre PL, Vaduganathan M, Claggett B, Packer M, Desai AS, Rouleau JL, et al. BNP during treatment with sacubitril/valsartan: PARADIGM-HF trial. J Am Coll Cardiol. 2019;73:1264–72. 10.1016/j.jacc.2019.01.018. 30846338 10.1016/j.jacc.2019.01.018 PMC7955687

[8] Mebazaa A, Davison B, Chioncel O, Cohen-Solal A, Diaz R, Maggioni AP, et al. STRONG-HF trial: up-titration of GDMT in acute heart failure. Lancet. 2022;400:1938–52. 10.1016/S0140-6736(22)02076-1. 36356631 10.1016/S0140-6736(22)02076-1

[9] Vaduganathan M, Claggett BL, Jhund PS, Cunningham JW, Pedrotty DM, Rizkala AR, et al. Estimating lifetime benefits of GDMT in HFrEF. Lancet. 2020;396:121–8. 10.1016/S0140-6736(20)30748-0. 32446323 10.1016/S0140-6736(20)30748-0

[10] Tromp J, Ouwerkerk W, van Veldhuisen DJ, Anker SD, Metra M, Samani NJ, et al. Network meta-analysis of GDMT in HFrEF. JACC Heart Fail. 2022;10:117–27. 10.1016/j.jchf.2021.09.004.

[11] Bao J, Kan R, Chen J, Zhai M, Zhang X, Liu J, et al. Combination pharmacotherapies for reverse remodeling in HFrEF. Pharmacol Res. 2021;169:105573. 10.1016/j.phrs.2021.105573. 33766629 10.1016/j.phrs.2021.105573

[12] Matsushita K, Delmas C, Marchandot B, Kibler M, Ohbe H, Sato N, et al. Optimal heart failure medical therapy and mortality in survivors of cardiogenic shock: insights from the FRENSHOCK registry. J Am Heart Assoc. 2024;13:e030975. 10.1161/JAHA.123.030975. 38390813 10.1161/JAHA.123.030975 PMC10944045

[13] Seferović PM, Polovina M, Adlbrecht C, Karavidas A, Lainscak M, Piepoli MF, et al. Navigating between scylla and charybdis: challenges and strategies for implementing guideline-directed medical therapy in heart failure with reduced ejection fraction. Eur J Heart Fail. 2021;23(12):1999–2007. 10.1002/ejhf.2378. 34755422 10.1002/ejhf.2378

[14] Dimond MG, Rosner CM, Lee SB, Li C, Doran JM, Khalid N, et al. Guideline-directed medical therapy implementation during hospitalization for cardiogenic shock. ESC Heart Fail. 2024;11:60–70. 10.1002/ehf2.14863. 10.1002/ehf2.14863 PMC11769606 39327768

[15] Thiele H, Zeymer U, Akin I, Jobs A, Fuernau G, de Waha-Thiele S, et al. Extracorporeal life support in infarct-related cardiogenic shock. N Engl J Med. 2023;389:1286–97. 10.1056/NEJMoa2307227. 37634145 10.1056/NEJMoa2307227

[16] Matsukawa R, Okahara A, Tokutome M, Ouchi K, Nishihara T, Nomura A, et al. A scoring evaluation for the practical introduction of guideline-directed medical therapy in heart failure patients. ESC Heart Fail. 2023;10:3352–63. 10.1002/ehf2.14524. 37671603 10.1002/ehf2.14524 PMC10682854

[17] Khan MS, Chan PS, Sherrod CF, Ahmed MI, Butler J, DeVore AD, et al. A generalizable approach to quantifying guideline-directed medical therapy. Circ Heart Fail. 2024;17:e011164. 10.1161/CIRCHEARTFAILURE.123.011164. 38742418 10.1161/CIRCHEARTFAILURE.123.011164 PMC11108743

[18] Asano T, Maeno Y, Nakano M, Kinoshita D, Tanaka Y, Hasegawa T, et al. Validation of a new scoring method to assess the efficacy of rapid initiation and Titration of combination pharmacotherapy for patients hospitalized with acute decompensated heart failure with reduced and mildly reduced ejection fraction. J Clin Med. 2024;13:2775. 10.3390/jcm13102775. 38792317 10.3390/jcm13102775 PMC11122539

[19] Tavares CAM, Azevedo LCP, Rea-Neto Á, Damiani LP, Costa ELV, Alexandre P, et al. Dapagliflozin for critically ill patients with acute organ dysfunction: the DEFENDER randomized clinical trial. JAMA. 2024;332:401–11. 10.1001/jama.2024.10510. 38873723 10.1001/jama.2024.10510 PMC11304119

