# Supplementary material for: Early initiation of guideline-directed medical therapy in patients with cardiogenic shock supported with Impella®: a two-center retrospective study
Source: BMC Cardiovasc Disord. 2025 Nov 19;25:818. doi: 10.1186/s12872-025-05311-5 (PMC12628905; doi:10.1186/s12872-025-05311-5)
Supplement: Supplementary file 1 — Supplementary Material 1 [file 12872_2025_5311_MOESM1_ESM.docx]

**Supplementary Table 1. The Simple Guideline-Directed Medical Therapy (GDMT) Scoring System**

| **Drug Class** | **Dose Level** | **Score** |
| --- | --- | --- |
| RASi (ACEi/ARB/ARNI) | None  ACEi/ARB (<50% of target dose)  ACEi/ARB (≥50% of target dose)  ARNI (Any dose) | 0  1  2  3 |
| Beta-blocker | None  <50% of target dose  ≥50% of target dose | 0  1  2 |
| MRA | None  Any dose | 0  2 |
| SGLT2i | None  Any dose | 0  2 |
| **Total** | — | **0–9** |

The scoring system proposed by Matsukawa et al. is based on the prescription and dosage of four key drug classes. The total score ranges from 0 to 9.

**Abbreviations:** RASi, renin–angiotensin system inhibitor; ACEi, angiotensin-converting enzyme inhibitor; ARB, angiotensin receptor blocker; ARNI, angiotensin receptor–neprilysin inhibitor; MRA, mineralocorticoid receptor antagonist; SGLT2i, sodium–glucose transporter 2 inhibitor.

**Supplementary Table 2.** **Baseline Characteristics and Outcomes Stratified by Etiology of Cardiogenic Shock**

|  | Non-ischemic (n=12) | | Ischemic (n=30) | *p* |
| --- | --- | --- | --- | --- |
| **Backgrounds** | | | | |
| Hospital A (%) | | 6 (50.0) | 9 (30.0) | 0. 29 |
| Hospital B (%) | | 6 (50.0) | 21 (70.0) |  |
| Age (years) | | 56.0 [49.8, 65.5] | 70.0 [62.5, 78.0] | <0.05 |
| Male sex (%) | | 10 (83.3) | 28 (93.3) | 0.57 |
| OHCA (%) | | 6 (50.0) | 5 (16.7) | <0.05 |
| Surgery (%) | | 4 (33.3) | 8 (26.7) | 0.72 |
| VA-ECMO (%) | | 7 (58.3) | 9 (30.0) | 0.16 |
| PVAD | | | | |
| Impella🄬 2.5/CP (%) | | 6 (50.0) | 26 (86.7) | <0.05 |
| Impella🄬 5.0/5.5 (%) | | 6 (50.0) | 4 (13.3) |  |
| SOFA | | 9.00 [6.8, 12.0] | 9.00 [5.00, 11.00] | 0.53 |
| APACHE II | | 23.5 [17.8, 27.3] | 19.00 [11.75, 24.25] | 0.23 |
| SBP (mmHg) | | 107.0 [96.5, 112.5] | 107.0 [92.0, 131.0] | 0.50 |
| DBP (mmHg) | | 71.0 [57.5, 80.5] | 77.0 [57.0, 96.0] | 0.89 |
| HR (bpm) | | 105.4±54.3 | 89.6±50.6 | 0.51 |
| EF (%) | | 29.62±13.81 | 29.29±11.89 | 0.95 |
| HFpEF (%) | | 1 (8.3) | 3 (10.3) | 1 |
| HFmrEF (%) | | 1 (8.3) | 2 (6.9) |  |
| HFrEF (%) | | 10 (83.3) | 24 (82.8) |  |
| **Comorbidity** | | | | |
| HT (%) | | 7 (58.3) | 18 (60.0) | 1 |
| DM (%) | | 4 (33.3) | 13 (43.3) | 0.73 |
| CKD (%) | | 2 (16.7) | 3 (10.0) | 0.61 |
| **Pre-hospital drug** | | | | |
| ACEi/ARB | | | | 0.63 |
| <50% of optimal dose | | 2 (16.7) | 2 (6.7) |  |
| ≥50% of optimal dose | | 3 (25.0) | 8 (26.7) |  |
| ARNI | | 2 (16.7) | 3 (10.0) |  |
| β-blocker | | | | 0.09 |
| <50% of optimal dose | | 3 (25.0) | 1 (11.1) |  |
| ≥50% of optimal dose | | 2 (16.7) | 4 (13.3) |  |
| MRA | | 4 (33.3) | 2 (6.7) | <0.05 |
| SGLT2i | | 5 (41.7) | 5 (16.7) | 0.11 |
| Pre-GDMT score | | 2.0 [0.0, 7.0] | 1.5 [0.0, 2.0] | 0.28 |
| **Laboratory data** | | | | |
| BNP (pg/mL) | | 180.5 [149.5, 361.5] | 260.0 [183.0, 523.0] | 0.46 |
| Cr (mg/dL) | | 1.2 [1.0, 1.7] | 1.3 [1.0, 1.6] | 0.76 |
| K (mmol/L) | | 4.3 [4.0, 4.5] | 4.3 [3.9, 4.8] | 0.83 |
| Hospital days | | 35.0 [31.0, 106.3] | 52.5 [34.5, 73.3] | 0.90 |
| **At discharge** | | | | |
| ICU days | | 14.5 [12.8, 19.3] | 12.0 [8.3, 24.0] | 0.38 |
| PVAD days | | 8.0 [6.8, 9.0] | 7.5 [4.0, 11.8] | 0.86 |
| SBP (mmHg) | | 119.3 ±16.5 | 101.2 ±17.4 | <0.05 |
| DBP (mmHg) | | 67.4 ±12.8 | 62.6 ±11.6 | 0.28 |
| HR (bpm) | | 80.8 ±12.4 | 77.1 ±15.1 | 0.48 |
| EF (%) | | 43.3 ±16.5 | 43.1 ±17.2 | 0.98 |
| HFpEF (%) | | 5 (50.0) | 9 (39.1) | 0.87 |
| HFmrEF (%) | | 1 (10.0) | 3 (13.0) |  |
| HFrEF (%) | | 4 (40.0) | 3 (13.0) |  |
| BNP (pg/mL) | | 146.0 [99.0, 714.0] | 260.0 [183.0, 523.0] | 0.60 |
| **Clinical outcomes** | | | | |
| Composite outcome | | 2 (16.7) | 9 (30.0) | 0.46 |
| HF event | | 1 (8.3) | 2 (6.7) | 1 |
| All-cause death | | 1 (8.3) | 7 (23.3) | 0.40 |
| GDMT score | | 5.5 [1.8, 8.0] | 6.0 [2.0, 8.0] | 0.81 |
| GDMT ≥5 | | 6 (50.0) | 19 (63.3) | 0.16 |

Data are presented as mean ± standard deviation for normally distributed variables, median [interquartile range] for non-normally distributed variables, and n (%) for categorical variables. P-values represent comparisons between the Non-ischemic and Ischemic groups.

**Abbreviations:** APACHE II, Acute Physiology and Chronic Health Evaluation II; ACEi, angiotensin converting enzyme inhibitor; ARB, angiotensin II receptor blocker; ARNI, angiotensin receptor–neprilysin inhibitor; BNP, brain natriuretic peptide; CKD, chronic kidney disease; Cr, creatinine; DBP, diastolic blood pressure; DM, diabetes mellitus; EF, ejection fraction; GDMT, guideline-directed medical therapy; HFmrEF, heart failure with mildly reduced ejection fraction; HFpEF, heart failure with preserved ejection fraction; HFrEF, heart failure with reduced ejection fraction; HR, heart rate; HT, hypertension; K, potassium; MRA, mineralocorticoid receptor antagonist; OHCA, out-of-hospital cardiac arrest; PVAD, percutaneous ventricular assist device; SBP, systolic blood pressure; SGLT2i, sodium-glucose co-transporter 2 inhibitor; SOFA, Sequential Organ Failure Assessment; VA-ECMO, veno-arterial extracorporeal membrane oxygenation

**Supplementary Figure 1.** **Survival Analysis Based on GDMT Score at Impella® Removal**


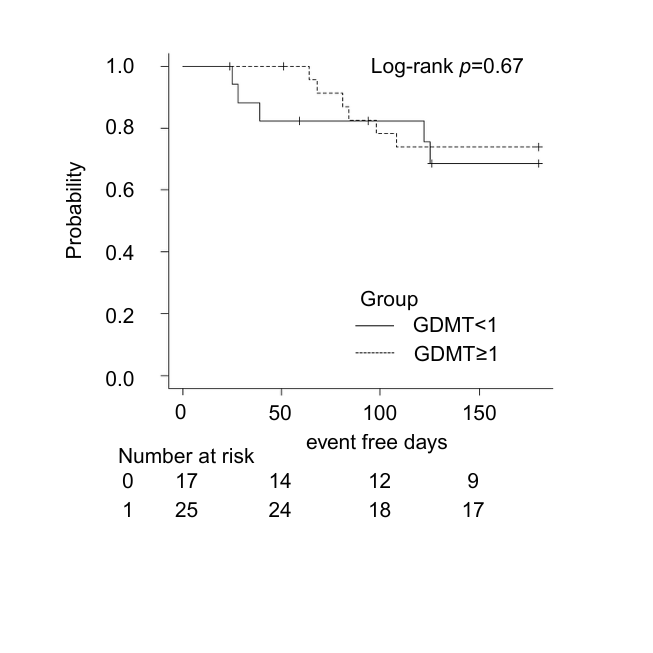


Kaplan–Meier curves illustrate 6-month event-free survival (a composite of all-cause mortality or heart failure readmission), comparing patients with a GDMT score <1 and those with a score ≥1 at the time of Impella® removal. No significant difference was observed between the two groups (log-rank test, *p* = 0.67)

**Supplementary Figure 2.** **Association Between GDMT Score at Impella® Removal and at Hospital Discharge**


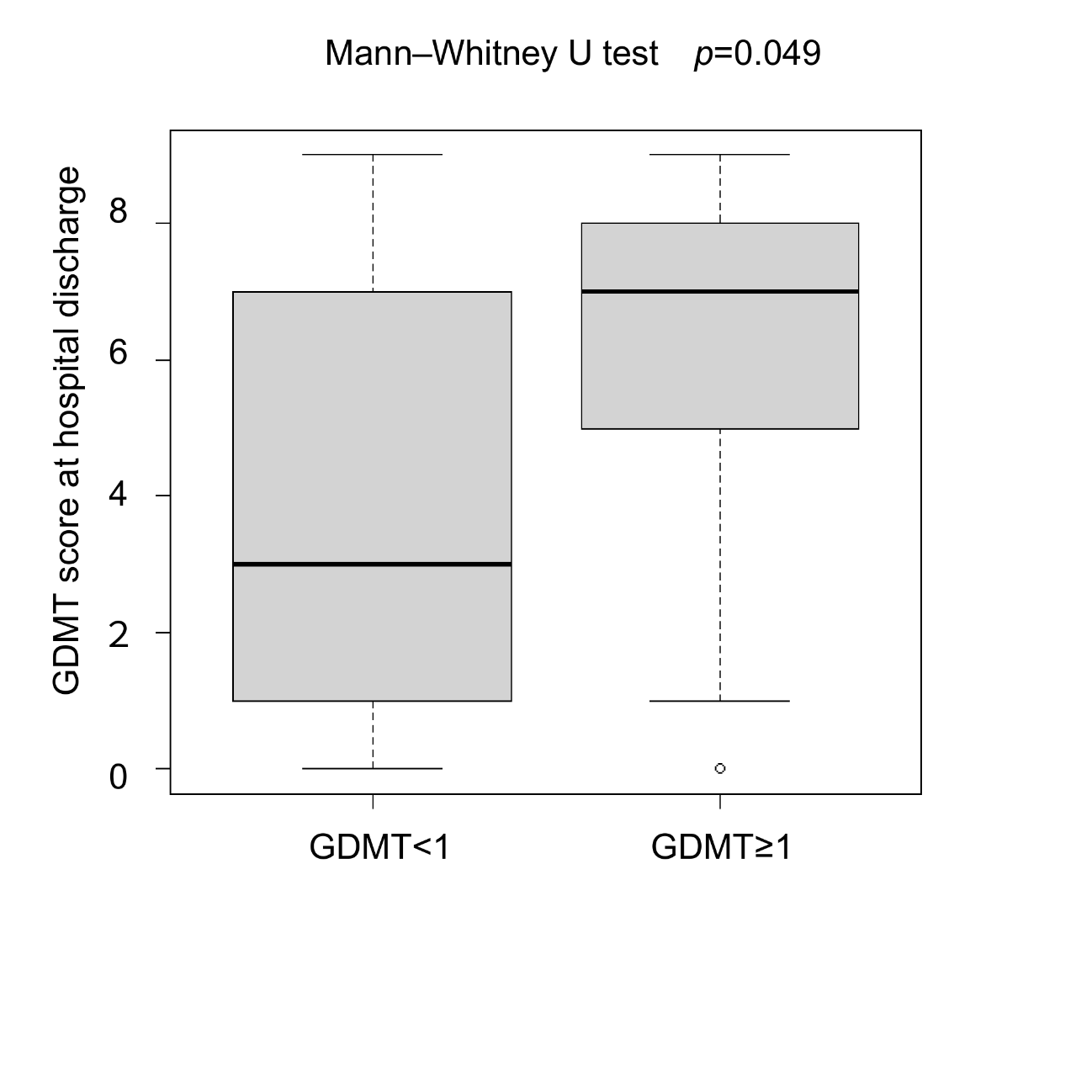


Comparison of GDMT scores at hospital discharge was conducted between patients with a low GDMT score (<1) and those with a high GDMT score (≥1) at the time of Impella® removal. Data are presented as boxplots showing the median, interquartile range, and minimum/maximum values. Statistical analysis was performed using the Mann–Whitney U test; *p* = 0.049.
